# Statistical analysis of Cotton-Jute blended ratio for producing good quality blended yarn

**DOI:** 10.1016/j.heliyon.2024.e25027

**Published:** 2024-01-19

**Authors:** Md. Redwanul Islam, Fahmida-E- Karim, Ayub Nabi Khan

**Affiliations:** a Department of Textile Engineering, Ahsanullah University of Science and Technology (AUST), Bangladesh; b Department of Textile Engineering, BGMEA University of Fashion & Technology (BUFT), Bangladesh

**Keywords:** Cotton-Jute, Blending, YQI, FQI, Regression, ANOVA

## Abstract

The present world is focusing on sustainable products. Most of the natural products collected from are environmentally friendly. In the textile sector the main raw material is fiber. Most textile products are made from the natural cotton fibers. But because of the shortage of this fiber, most of the researchers are looking forwards to other sources of natural fibers. Here in Bangladesh the natural jute fiber is available and the textile industries are making jute products but the jute products are comparatively lower price than cotton products. That's why some factories are making cotton-jute blended yarn to minimise the cost and increase the product's quality and appearance. Here in this research work, it was tried to identify the best cotton-jute blended ratio for producing good quality yarn. 80C-20 J, 60C-40 J and 40C-60 J blended ratios are compared with 100 C and 100 J yarn to analyse the results. The CV m%, Thick/Km +50 %, Neps/Km +200 %, IPI, RKM and Elongation% of blended yarns are evaluated and compared the results between the ratios. After that the yarn quality index (YQI) was calculated to identify the ratio which indicates a relation between yarns strength, elongation% and CVm. The other quality index was fiber quality index (FQI) which indicates a relation between fibers strength, fiber mean length, elongation% and fiber fineness. One way ANOVA was applied to see the significance level between the independent variables. Box plot was applied to see the visual effect of statistical analysis at the same time the regression results show the impact of cotton-jute ratio with an equation, through which it was easy to identify the perfect ratio. It was found that higher percentage of cotton and lower percentage of jute fiber blended yarn shows good results than others. The products which were made from the ratios were shown good results for their different use of purposes.

## 1 Introduction

Cotton is equivalent to white gold. It is used for a number of applications, but most notably in the production of textiles utilized in the production of a substantial amount of men's clothes. Cotton demand continues to rise as the world population grows. It is suitable for light weight apparel textiles. Cotton fibers are single-celled outgrowths of individual epidermal cells on the exterior integument of growing cotton fruit ovules. Cotton fibers elongate dramatically throughout development [[1], [2], [3]]. Cotton fiber is the most common natural raw material used in the textile industry, and it is one of the world economy's cornerstones. Cotton is a member of the Malvaceae family and belongs to the genus Gossypium. Fiber development is divided into four overlapping phases (initiation, extension, secondary membrane synthesis, and maturity) according on the number of days post-anthesis [4,5]. Most evolutionary change has an unknown genetic basis. The cotton seed trichome, found in all 50 species of the genus Gossypium and commonly known as “cotton fiber” in domesticated species, is an uncommon example of evolutionary innovation using a single-celled structure [6,7].

Jute fiber has been extracted from plants of the genus Corchorus of the Malvaceae family. Jute is a lignocellulosic fiber that is both a textile fiber and a wood fiber. It is classified as bast fiber (fiber derived from the plant's bast or skin). Jute fiber is composed of cellulose (64.4 %), hemicellulose (12 %), pectin (0.2 %), lignin (11.8 %), water soluble (1.1 %), wax (0.5 %), and water (10 %). Jute fiber is made up of many cells. These cells are made up of crystalline cellulose microfibrils that are linked to a full layer by amorphous lignin and hemicellulose [8,9]. Jute is one of the cheapest natural fibers, second only to cotton in terms of production, and its fibers are essentially formed of the plant components cellulose and lignin. Jute is referred to as the “golden fiber” because of its golden brown hue and significance. Jute is the most environmentally beneficial fabric, from seed to expired fiber, because the expired fibers may be recycled several times. Jute fiber is strong, inexpensive, long-lasting, and versatile. It may be used to manufacture hessian bags, garden twine, ropes, and carpets, among other things. In terms of textile fiber output, jute is second only to cotton. It is a significant textile fiber as well as a raw material for non-traditional and value-added goods [[8], [9], [10], [11]].

Natural fibers vary in their fundamental physical, mechanical, and surface qualities. As a result, selecting a combination of machineries and machine settings for processing of natural fiber blends to achieve required yarn quality at the lowest cost necessitates a thorough understanding of the qualities of fibers as well as a high level of expertise in their processing technologies. Jute and cotton are both considered natural fabrics. As a result, jute-cotton mixed yarn absorbs all natural fiber qualities. Jute-cotton (jutton) mixed yarn is soft to the touch, has a low electrostatic charge, is hygroscopic, and has high insulating qualities. As a result, jutton are widely used in household textiles, floor coverings, curtains, shoes, handicrafts, and other applications [12,13]. Blending multiple fabric types is a typical way to improve a garment's performance, aesthetic, and utilitarian features. Material type, mix ratio, yarn specification, and machine type and settings all influence the quality and cost of blend yarn [14,15].

One research work deals with cotton-jute blending ratios 80C:20J, 65C:35J and 50C:50J for producing denim fabric. This research shows that lower value of jute fiber shows the good results [36]. Another research work was about the analysis of physical and chemical properties of Cotton Jute blended denim after a sustainable (Industrial Stone Enzyme) wash that was the focus of this paper. Following the washing procedure, the EPI, PPI, and GSM rose. The warp strength fell slightly in this work, although not much. The weft strength, on the other hand, was somewhat enhanced. It discovered a new coating via this process that will serve to enhance the charm of the denim. Here the researchers used 75C:25J blended ratio [37]. Furthermore another research work is about the jute-cotton blended fabric was evaluated based on weave design, yarn count, fabric density, cover factor, weight per unit area (g/m2), fabric strength, dyeing performances such as wash and rubbing fastness, reflectance, and color strength for samples A (50:50) and B (30:70). According to the findings, the properties of mixed yarn fabric may be utilized as entirely cotton fabric, potentially reducing reliance on importable cotton fiber [38].

## 2 Materials and methods

### 2.1 Materials

In this study cotton (Mali) and jute fiber (Faridpur, Bangladesh) have been used for making cotton-jute blended yarn. The blending ratios were 80C-20J, 60C-40J and 40C-60J but the yarn produced from these was fixed, that was 20 Ne.

Here Table 1 indicates the fibers properties of cotton and jute fiber and Table 2 shows the physical properties of cotton, jute as well as different blended yarn physical properties, which will be used for statistical analysis.

**Table 1:** Fiber properties.

Fiber	UHML (mm)	Strength (gm./tex)	Elongation (%)	SFI	MIC (micro gm./inch)	FQI
Cotton (Mali)	32.11	39.61	6.65	7.2	4.23	2001.95
Jute (Bangladesh)	110.3	49.56	1.84	22.3	8.34	1197.7

**Table 2:** Physical properties of yarn.

Blend composition	CVm%	Thin/Km −50 %	Thick/Km +50 %	Neps/Km +200 %	IPI	RKM	Elongation (%)
100C	13.8	0.6	75.6	97.4	173.6	18.3	6.3
80C:20J	18.5	18.2	603.6	907.3	1529.1	17.5	8.45
60C:40J	21.3	130.43	1412	1890.4	3432.83	14.3	6.1
40C:60J	29.4	2043	2958	5163.7	10164.7	13.4	4.3
100J	32.3	2989	1850	5949.2	10788.2	28.23	2.19

### 2.2 Methods

For producing prefect blended ratio, all the parameters from carding to ring frame were constant for making a particular count from different cotton-jute blended ratios. Table 3 shows the parameters which were used as constant for all blended ratios. Mainly the blending was done at draw frame for getting the perfect blended ratio. Because perfect blending can be found from draw frame. Draw frames reduce length and thickness discrepancies by aligning the fibers parallel to one another and evenly mixing various fiber kinds. This procedure not only increases the strength and longevity of the yarn, but it also paves the way for other procedures that will be more effective [16,17].

**Table 3:** Machines parameters settings.

Machines	Model	Del. Speed (m/min)	Draft	Count (Hank)	Doubling ratio	Spindle speed (rpm)	TPI	Efficiency%
Carding	TC-06	215	92	0.01	–	–	–	93
Drawing	SB-D40	550	10	0.11	8:1	–	–	87
Simplex	FL 200	22.41	10	1.0	–	900	1.02	80
Ring frame	F1520	18.4	20	20	–	9500	13.12	90

After making the blended yarn from ring frame machine, the samples are needed to be checked to analyse the quality of blended yarn. Table 4 indicates the testing parameters according to the testing standards. Here CVm%, Thick, Thin, Neps, IPI, RKM and Elongation% have been measured for samples. Ten samples for each blended ratio were produced for statistical analyse. The term “Coefficient of Variation of the Mean” (CVm%) refers to a measurement that expresses variance in a group of data points as a proportion of the mean value of the data set. The evenness or uniformity of yarn thickness or diameter is frequently evaluated using CVm% in the context of yarn manufacturing [22]. Irregularities properties like “Thick,” “Thin,” “Neps,” and “IPI” (Imperfections per Unit Length) are the most significant things in yarn. Thick and thin means differences in yarn diameter that affect the yarn strength. Neps, which impair the strength and texture of yarn, are tangled fiber masses created by short fibers or contaminants. IPI, which includes neps, thick-thin variances, and other flaws, quantifies total faults in a certain yarn length. For producing high-quality yarn, these anomalies must be managed, which is achieved through complicated processes and strict quality control techniques [23]. So the lower value of these indicates the good quality of yarn.

**Table 4:** Parameters and standards of testing.

Parameters measured	Name of testing instrument	Testing standard
Yarn Unevenness (CVm%, Thick, Thin, Neps, IPI)	HVI 1000	ASTM D1445 [18], ASTM D1447 [19] and ASTM D 1448 [20]
Yarn tensile properties (RKM, Elongation %)	AUTODYN II single (Mesdan Lab) Lea strength tester	ASTM D1578-93(2016) [21]

The abbreviation RKM stands for “Reisskilometer” or “Breaking-kilometre”. The “breaking force of yarn per kilometres"—the speed at which yarn will snap under its own weight—can be used to represent this. In g/tex, this is the same as breaking load [24,25]. The term “elongation %" also known as “elongation at break” or “Percent Elongation,” is frequently used. It is an important indicator that offers insights into the flexibility, strength, and tension behaviour of the yarn in the context of yarn manufacturing [26]. So the hogher value of these indicates the good quality of yarn.

#### 2.2.1 Yarn quality index (YQI) and fiber quality index (FQI)

Yarn quality is often determined by one factor, namely its strength. Three parameters—strength, elongation, and CVm—have been included in the researchers' creation of a yarn quality index (YQI) [28,29]. In the textile business, cotton fiber quality is measured using the Fiber Quality Index (FQI). It offers a comprehensive numerical representation of several fiber characteristics that are crucial for deciding if cotton is suitable for yarn. It depends on fiber strength, mean length, elongation and Fineness [27,30]. [27,27].

(i) $$YQI=\frac{Strength\times Elongation}{CVm}$$

(ii) $$FQI=\frac{Strength\times Mean\;length\times Elongation}{Finenness}$$

From the equation (i) and (ii) the YQI and FQI have been measured and these values indicate the quality of yarn with respect to fiber quality.

#### 2.2.2 Boxplot diagram

Boxplot is a visual representation of variation in a set of data. A histogram analysis is generally sufficient, but a boxplot can provide additional information while allowing the display of many sets of data on the same graph. Boxplots may aggregate data from several sources and show the results in a single graph, making them extremely efficient and easy to read. Boxes make it easier to compare data from different categories. Tables, charts, and graphical plots are some forms of graphically communicating summary statistics. Graphical plots are appealing because they convey a lot of information in a succinct and graphical manner, allowing for quick examination and interpretation of the data [31,32].

**Fig. 1:**
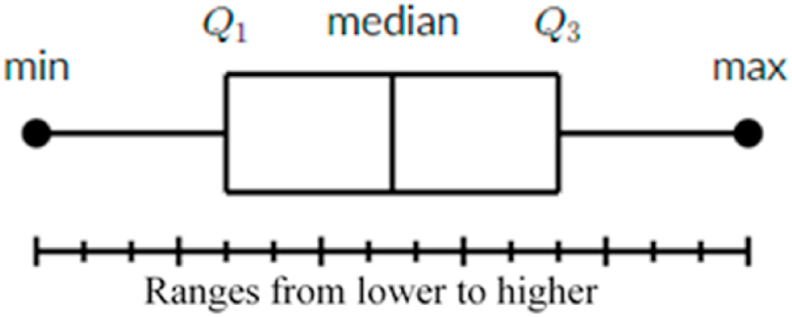
Basic structure of box plot diagram.

From Fig. 1 the boxplot synopses data are based on the median and correspond to the smallest observation, the median of the first half of the data (first quartile, Q1), the median of the second half of the data (second quartile, Q2), the median of the third quartile (Q3), and the biggest observation. The interquartile range (IQR) is the region between the first and third quartiles that indicates data dispersion (IQR = Q3 - Q1). The IQR corresponds to the lone box in the presentation and covers roughly half of the observations closer to the median. The smallest and biggest observations are those that lie beyond the lines that link the IQR to the smallest or largest value [33].

#### 2.2.3 Regression

The link between two variables is also revealed using regression. In contrast to correlation, regression considers one variable as a result (dependent variable) and the other as a predictor variable. Before estimating the unknown variable (dependent variable), regression analysis evaluates the nature of the link between two or more variables using known components (independent variables). There are two types of variables in a regression analysis. The variables used to predict the variable of interest are known as independent, explanatory, or predictor variables, whilst the variable whose value is to be predicted is known as the dependent, explained, or regressed variable [34,35].

(iii) Y=α+βX+e

here the equation (iii) indicates the following information,

Y = dependent variable; α = intercept (constant amount); β = coefficient of independent variable; X = independent variable; e = error or the ‘noise’ term that reflect other variables to have an effect on Y.

#### 2.2.4 Flowchart of working procedure

**Fig. 2:**
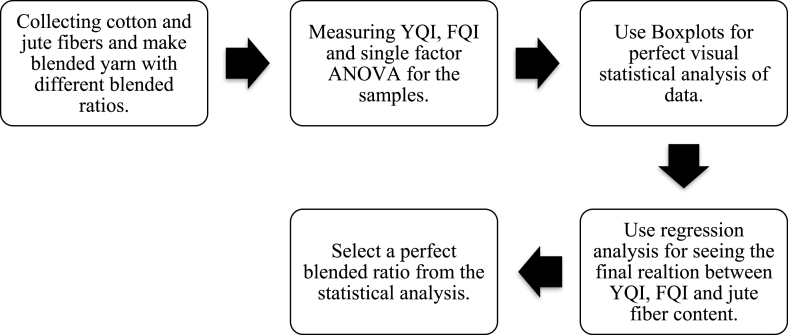
Flowchart of the whole work.

Fig. 2 highlights the whole working process of this research work. This summarize the working process from raw materials collection to perfect blended ratio selection.

## 3 Results and discussions

The present study has aimed primarily to analyse the effect of blend ratio on cotton-flax blended yarns properties and focus on optimal blend ratio. The yarn test report is summarized in Table 1, Table 2 summarized the repot of cotton and jute fibres.

### 3.1 YQI and FQI analysis

Table 5 summarize the yarn quality index of cotton-jute blended yarn as well as fibre quality index. Here shows YQI is higher for pure cotton made yarn and then it gradually decrease with the increase of the percentage of jute fiber content. It shows less YQI for pure jute made yarn. It is known to us that YQI depends on the strength, elongation and CVm of the yarn. For jute the elongation is less than the cotton and CVm is larger than cotton. That's why the YQI is lower for jute yarn. Again it also indicates that FQI is higher for pure cotton and it gradually decrease with the increase of jute fiber.

**Table 5:** YQI and FQI.

Index	Parameter	100C	80C:20J	60C:40J	40C:60J	100J
YQI	Mean	8.4	7.9	4.4	1.93	2.09
FQI	Mean	2001.95	1907.248	1638.04	1220.16	1197.7

ANOVA is a powerful and strong statistical approach that is important for studying several groups or categories. The one-way ANOVA can tell you whether or not there are significant differences in your independent variables. Table 6 indicates the single factor ANOVA between the groups of 100C, 80C:20J, 60C:40J, 40C:60J and 100J. Again also analyse the effect of significant within the groups. Sum of square of between the groups is 193.9574 and within the groups is 0.62996. The F crit value is very low, which is about 2.866081. Lower the F crit value is good for the results.

**Table 6:** ANOVA: Single factor.

Source of Variation	SS	MS	F	*P*-value	F crit
Between Groups	193.9574	48.48936	1539.443	1.39E-24	2.866081
Within Groups	0.62996	0.031498			

The *P*-value is 1.39E-24, which is less than 0.05. That means the results of different blended ratios which have been got are significant impact on the quality of yarn. The justification of statistical analysis also seen from the ANOVA table. Lower the value of *P*-value increase the acceptance of the data as well as justify the values. Here the *P*-value is too minimum which is close to zero, which means the values are justified.

The Post-HOC test in Table 7 of YQI was performed to identify blends that differ significantly in terms of YQI. For POST-HOC test the alpha level was 0.01667 (0.05/3) and every blended shows the significant level strongly.

**Table 7:** POST-HOC and ALPHA test.

POST-HOC test			ALPHA test	
Groups	*P*-value two tail (T-test)	Significant	Test	Alpha
80C:20J Vs 60C:40J	4.71E-07	Yes	ANOVA	0.05
80C:20J Vs 40C:60J	8.33E-09	Yes	POST-HOC	0.01667
60C:40J Vs 40C:60J	6.12E-05	Yes		

### 3.2 Boxplot analysis

This Boxplot analysis has been applied for visual analysis of statistical data for different cotton-jute blended ratios.

#### 3.2.1 Yarn unevenness analysis

**Fig. 3:**
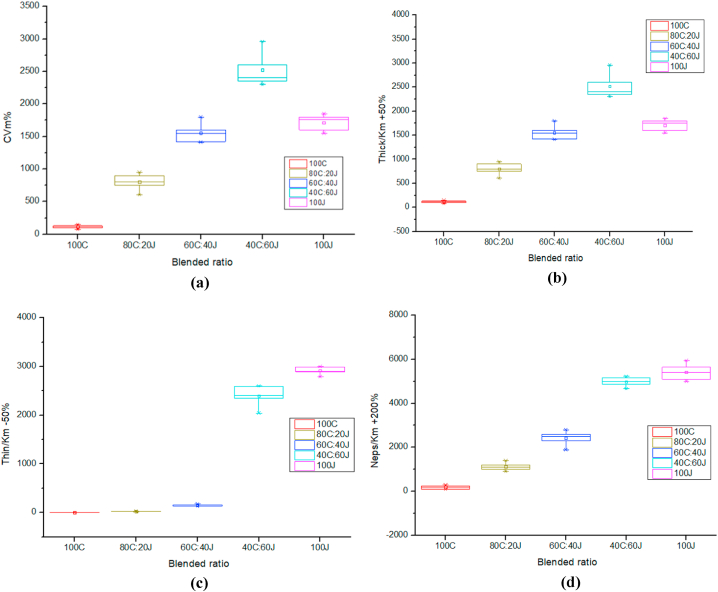
Effect of blended ratio on (a) CVm %; (b) Thick/Km +50 %; (c) Thin/Km −50 % and (d) Neps/Km +200 %.

The CVm%, thick, thin and neps indicates the unevenness of yarn. Fig. 3 shows the boxplot diagram of these unevenness parameters. Fig. 3 (a) indicates with the increase of jute percentage in blending ratio the CVm% is increased and it slightly decrease at pure jute yarn.

Fig. 3 (b) shows same result as Figure (a). In Fig. 3 (c) highlights the thin values of different ratios, where the percentage of thin with the increase of jute fiber and it shows maximum limit at pure jute yarn. Fig. 3 (d) shows same result as Fig. 3 (c).

**Fig. 4:**
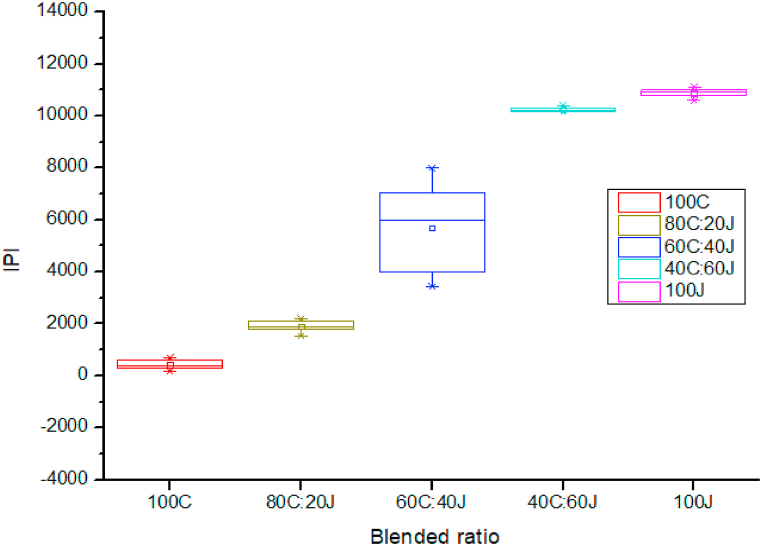
Effect of blended ratio on IPI.

Fig. 4 indicates IPI values with respect to different blending ratios. IPI is the summation of thick, thin and neps of a yarn. Hers it shows the IPI vale increase with the increase of jute fiber percentage in the yarn. It means yarn unevenness increase with the increase of jute fiber content.

#### 3.2.2 Yarn tensile properties analysis

**Fig. 5:**
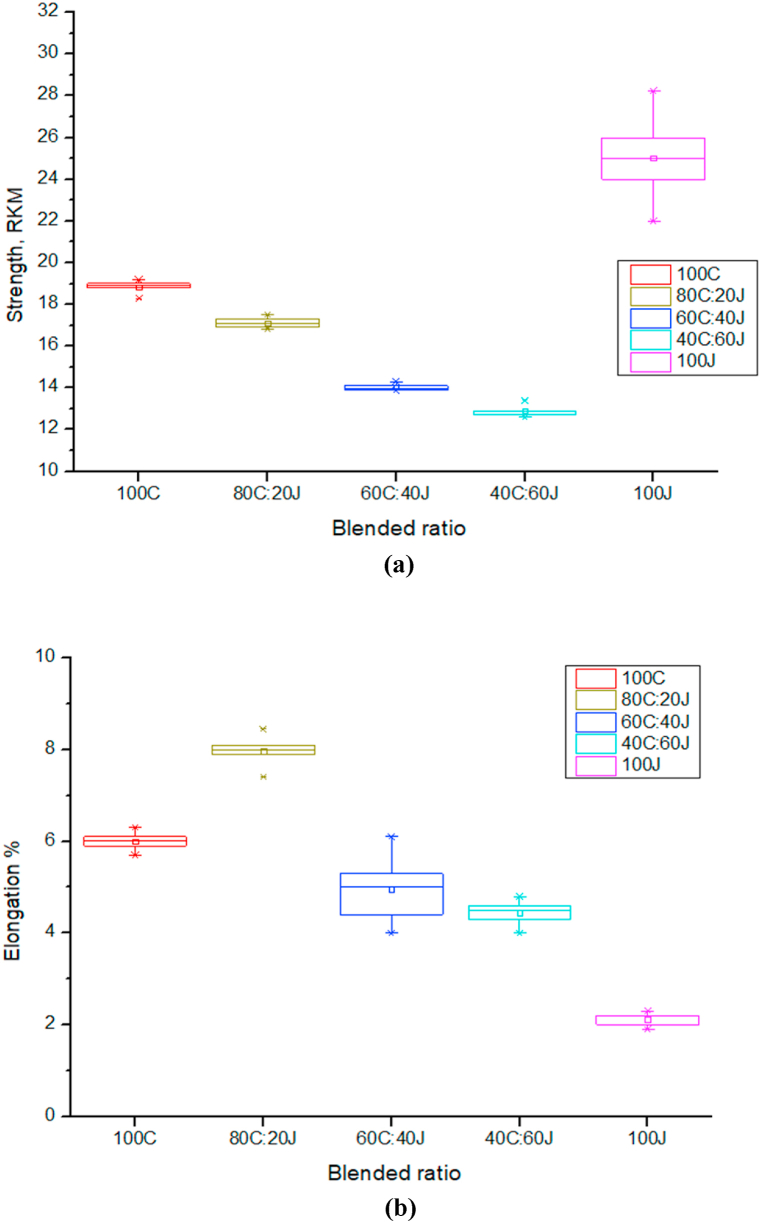
Effect of blended ratio on (a) strength (RKM) and (b) elongation %.

Fig. 5 (a) shows that the strength of yarn decrease with the increase of the percentage of jute. But it shows higher strength than cotton at stage of 100 % jute fiber. It means jute fiber strength is much more that cotton fiber. Fig. 5 (b) indicates the elongation%, here 80C:20J ratio shows higher elongation than 100 % cotton. But after that it decrease with increase of the percentage of jute. It means jute fiber shows less elongation than cotton.

### 3.3 Regression analysis

Regression analysis is used to identify the correlation between the dependent and independent variables. Here Table 8 summarize the regression result for YQI with respect to FQI and jute fiber content. For FQI the *P*-vale is 3.04E-18, which means the FQI significantly affects YQI. At the same time the *P*-value for only jute content is 1.1E-09, which is also significantly influence the quality of yarn.

(iv) YQI = 8.170865–0.0784*Jute Content

(v) YQI = −8.22069 + 0.008255*FQI

**Table 8:** SUMMARY OUTPUT: regression analysis.

Regression Statistics		ANOVA Statistics				Coefficient Statistics	
Multiple R	R Square	SS	MS	F	*P*-value	Intercept	Independent variable
0.982336	0.963462	187.7739	187.7739	633.8546	3.04E-18	−8.22069	FQI = 0.008255
0.89835	0.798643	157.0384	157.0384	96.19113	1.1E-09	8.170865	Jute content = −0.07284

**Fig. 6:**
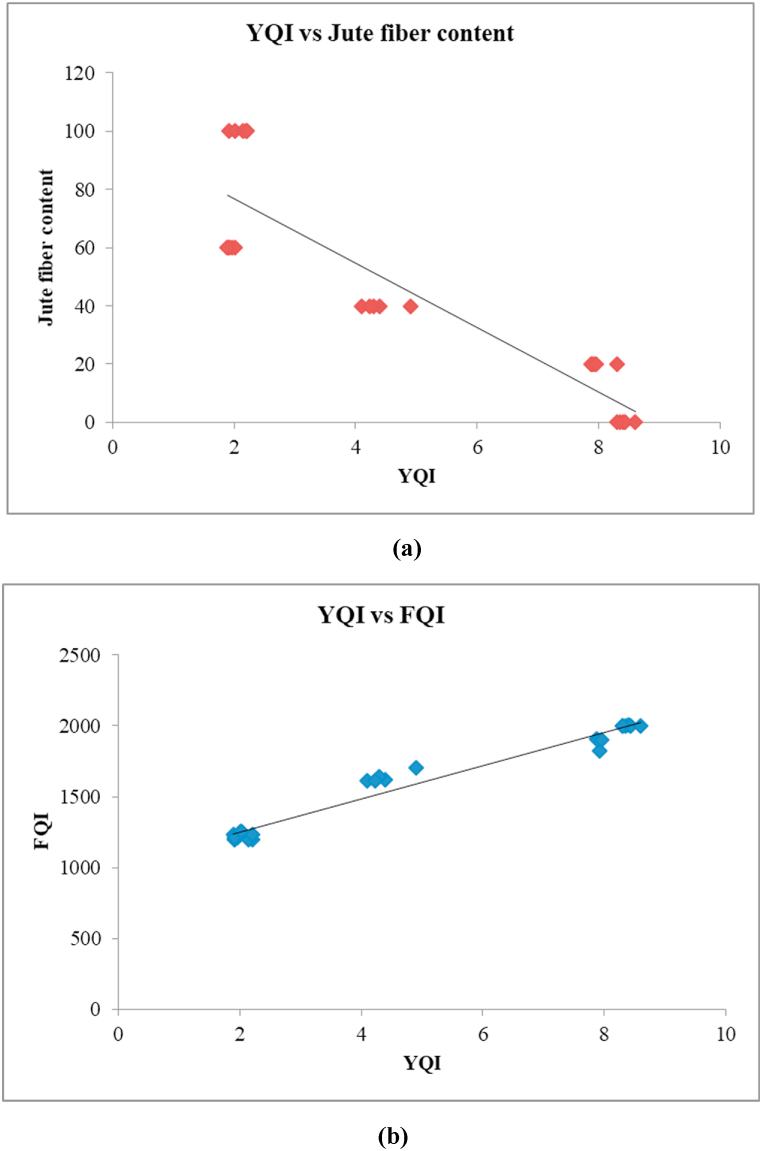
Regression effect of YQI with (a) jute fiber content and (b) FQI.

Equation (iv) is the regression analysis outcome between YQI and jute fiber content. The multiple R and R square of it are 0.89835 and 0.798643. Which are close to one that means the values are accepted for data analysis. Fig. 6 (a) is the graph of this equation. Here this is clearly seen that with the increase of jute fiber content the YQI decrease. Equation (v) is the regression analysis outcome between YQI and jute FQI. The multiple R and R square of it are 0.982336 and 0.963462. Which are close to one that means the values are strongly accepted for data analysis. Fig. 6 (b) is the graph of this equation. Here this is clearly seen that with the increase of FQI the YQI decrease.

## 4 Conclusion

This work aim was to statistical identify the perfect blended ration for cotton-jute blended yarn. For the statistical analysis CVm%, thick, thin, neps, IPI, RKM, elongation% were analysed for selecting the perfect ration. Here 80C:20J, 60C:40J and 40C:60J blended yarns have been used and the data are compared with 100C and 100J. The YQI and FQI was measured for perfect influence of blended ratio on yarn. From the statistical analysis it has been shown that yarn quality decrease with the increase of jute fiber percentage and at the same time yarn quality increase with the increase of FQI. For jute fiber the FQI is lower than cotton and it gradually decrease with the increase of jute fiber ratio. The boxplot analysis also indicates the yarn unevenness increase with the increase of jute fiber portion. At the same time it also highlighted that the tensile properties also decrease with the increase of the jute fiber. So Lower percentage of jute fiber in the blended ratio is perfect for cotton-jute yarn blended yarn and that is 80C:20J. This ratio shows optimum results for all kinds of analysis. So here it can be concluded that lower percentage of jute fiber in the cotton-jute blended yarn is perfect for good quality yarn. The advantage of the research work is through this work anyone can gather overall average idea about different cotton-jute blended ratios and their applications. The applications of the products of these ratios also help to find out which final products should need to be produced by using which ratio.

## 5 Applications

Table 9 deals with the applications of Cotton-Jute, which ratios have been used in the research work. The ratios are serial gradually with higher strength to lower strength. For perfectly comparing the strength of the products, on an average the same dimensions products were collected with their proper applications.

**Table 9:** Applications of 100J, 100C, 80C:20J, 60C:40J and 40C:60J ratios blended yarns.

Blended ratios	Images of the products	Carrying Load (Kg)	Applications
100J		5 Kg	Carrying rice.
100C		3 Kg	Carrying vegetables.
80C:20J		2 Kg	Carrying heavy documents.
60C:40J		1.5 Kg	Carrying laundry products.
40C:60J		Around 1 Kg	Carrying office tiffin.

The applications of the products from different ratios meet the strength of the research work in Fig. 5. Where it shows 100J has higher strength and 40C:60J has the lower strength. The applications of the products also show the same results, which justify the assumption on the selection of cotton-jute blended ratios.

### 5.1 Correlate blended yarn properties with fabric properties

The input materials of different ratios of cotton-jute blended yarns properties are compared with the products produced from those blended yarns and compared the results to see the correlation between them.

**Fig. 7:**
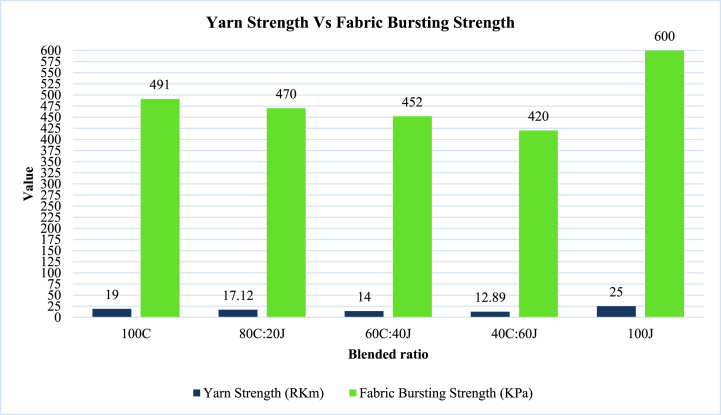
Yarn strength Vs fabric bursting strength.

Table 10. & Fig. 7. Highlights the mean values of yarn strength (RKm) and fabric bursting strength (KPa). These table and figure clearly correlate Table 9. & Fig. 5 (a).

**Table 10:** Yarn strength Vs fabric bursting strength.

Blended ratios	Yarn Strength (RKm)	Fabric Bursting Strength (KPa)
100C	19	491
80C:20J	17.12	470
60C:40J	14	452
40C:60J	12.89	420
100J	25	600

From Table 10. & Fig. 7. This is clearly seen that the 100J blended ratio yarn shows the higher yarn strength than others and the fabric produced from this yarn shows higher bursting strength than others. The strength of blended yarn gradually decrease like 100C, 80C:20J, 60C:40J and 40C:60J. At the same time the fabrics produced from these ratios show the bursting strength according to the same order as blended yarn.

**Fig. 8:**
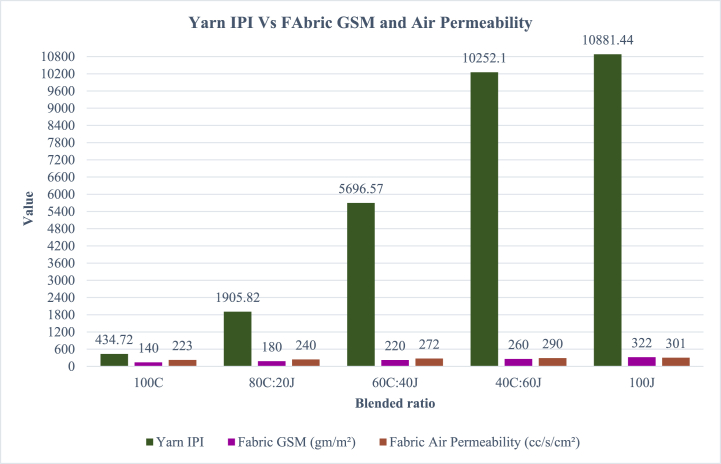
Yarn IPI Vs FAbric GSM and air permeability.

Table 11. & Fig. 8. Summarize the mean values of blended yarn IPI which compared with the fabric GSM (gm/m^2^) and air permeability (cc/s/cm^2^) values.

The GSM value of fabric is affected by the IPI (thick + thin + neps) value. Higher the IPI value in the yarn increases the areal density of the fabric. Here the areal density GSM (gm/m^2^) of fabric is evaluated to correlate the value with yarn IPI. Another fabric property is air permeability, which is also affected by GSM of fabric. Higher the GSM value increases the air permeability of the fabric. So the IPI of yarn is correlated with the fabric GSM and air permeability property.

Table 11. & Fig. 8. Clearly correlate with Fig. 4. Which is about the IPI of values of different blended ratios of yarns. The IPI value of 100J jute is higher than other blended ratios of yarns. The IPI values of yarn gradually decrease like 40C:60J, 60C:40J, 80C:20J and 100C. At the same time the products produced from these shows the same order for fabric GSM and air permeability property. The fabric shows higher GSM and air permeability properties for 100J and gradually decreases as IPI values of blended yarn.

**Table 11:** Yarn IPI Vs FAbric GSM and air permeability.

Blended ratios	Yarn IPI	Fabric GSM (gm/m^2^)	Fabric Air Permeability (cc/s/cm^2^)
100C	434.72	140	223
80C:20J	1905.82	180	240
60C:40J	5696.57	220	272
40C:60J	10252.1	260	290
100J	10881.44	322	301

So by comparing the values of blended yarns properties with the properties of the products made from these blended yarns, it is seen that the values are completely correlated with each other. It means there is correlation between input material (yarn) and out material (fabric).

## 6 Limitations and assumptions

The research work was done in a factory with highly request. It was thought that the work will become very difficult to analyse these different types' blended ratios in any factory but it was matter of joy that the factory allows us to do that changes while there production was running. The number of machines were limited that's why it took a long time to get the desire product. So it was quite difficult to produce yarn with more blended ratios, that's why the average ratios were taken to get the overall scenario of the result. For this research work there was no funding, so it was very difficult to do the whole research work without any funding.

## 7 Acceptability of the results

After collected the different ratios blended yarns the products which haven made with these ratios were searched and found that the products which were made from 100J were used for carrying heavy load than other ratios like 100C, 80C:20J, 60C:40J and 40C:60J. The practical use of these with figure are discussed in the applications and the correlation analysis of the properties of the yarns and properties of the fabrics made from those blended ratios also increase the acceptability of the results.

## Funding statement

This research did not receive any fund.

## Additional information

No additional information is available for this paper.

## Data availability statement

Data will be made available on request.

## CRediT authorship contribution statement

**Md Redwanul Islam:** Writing – review & editing, Writing – original draft, Visualization, Validation, Supervision, Software, Formal analysis. **Fahmida-E- Karim:** Project administration, Methodology, Investigation. **Ayub Nabi Khan:** Methodology, Investigation.

## Declaration of competing interest

The authors declare that they have no known competing financial interests or personal relationships that could have appeared to influence the work reported in this paper.

## References

[1] Basra A.S., Malik C.P. (1984 Jan 1).

[2] Anderson D.B., Kerr T. (1938 Jan). Growth and structure of cotton fiber. Ind. Eng. Chem..

[3] Qin Y.M., Zhu Y.X. (2011 Feb 1). How cotton fibers elongate: a tale of linear cell-growth mode. Curr. Opin. Plant Biol..

[4] Ji S., Lu Y., Li J., Wei G., Liang X., Zhu Y. (2002 Sep 6). A β-tubulin-like cDNA expressed specifically in elongating cotton fibers induces longitudinal growth of fission yeast. Biochem. Biophys. Res. Commun..

[5] Qin Y.M., Zhu Y.X. (2011 Feb 1). How cotton fibers elongate: a tale of linear cell-growth mode. Curr. Opin. Plant Biol..

[6] Hovav R., Udall J.A., Chaudhary B., Hovav E., Flagel L., Hu G., Wendel J.F. (2008 Feb). The evolution of spinnable cotton fiber entailed prolonged development and a novel metabolism. PLoS Genet..

[7] Hu Y., Chen J., Fang L., Zhang Z., Ma W., Niu Y., Ju L., Deng J., Zhao T., Lian J., Baruch K. (2019 Apr). Gossypium barbadense and Gossypium hirsutum genomes provide insights into the origin and evolution of allotetraploid cotton. Nat. Genet..

[8] Sanyal T., Sanyal T. (2017).

[9] Ali A., Shaker K., Nawab Y., Jabbar M., Hussain T., Militky J., Baheti V. (2018 May). Hydrophobic treatment of natural fibers and their composites—a review. J. Ind. Textil..

[10] Sanyal T., Sanyal T. (2017).

[11] Kiron M.I. (2012). https://textilelearner.net/features-properties-and-uses-of-jute-fiber/.

[12] Basu G., Roy A.N. (2008 Mar 6). Blending of jute with different natural fibres. J. Nat. Fibers.

[13] Hasan R., Nishi S.I., Mia R., Islam M.M., Hasan M.M., Ahmed F. (2023 Feb 1). Ecofriendly functionalization of jute–cotton blended yarn using Azadirachta Indica leaves. Environ. Technol. Innovat..

[14] Malik S.A., Tanwari A., Syed U., Qureshi R.F., Mengal N. (2012 Jul 1). Blended yarn analysis: Part I—influence of blend ratio and break draft on mass variation, hairiness, and physical properties of 15 tex PES/CO blended ring-spun yarn. J. Nat. Fibers.

[15] Malik S.A., Mengal N., Saleemi S., Abbasi S.A. (2013 Jul 3). Blended yarn analysis: Part II—influence of twist multiplier and back roller cot hardness on mass variation, hairiness, and physical properties of 15 tex PES/CO-blended ring-spun yarn. J. Nat. Fibers.

[16] Ray S., Ghosh A., Banerjee D. (2018 Jun). Analyzing the effect of spinning process variables on draw frame blended cotton mélange yarn quality. J. Inst. Eng.: Series E..

[17] Basu G., Roy A.N. (2008 Mar 6). Blending of jute with different natural fibres. J. Nat. Fibers.

[18] (2021). ASTM International - ASTM D1445/D1445M-12. https://standards.globalspec.com/std/14364120/ASTM%20D1445/D1445M-12(2021).

[19] (2021). Astm D1447-07. https://www.astm.org/d1447-07r21.html.

[20] (2023). Astm D1448-11. https://www.astm.org/d1448-11.html.

[21] (2023). ASTM International - ASTM D1578-93(2016). https://standards.globalspec.com/std/3860484/ASTM%20D1578-93(2016).

[22] (2023). Unevenness and coefficient of variation of yarn. https://textilestudycenter.com/unevenness-coefficient-variation-yarn/#google_vignette.

[23] (2023). Imperfection index of yarns (IPI) | Reasons for increasing Imperfection index of yarn. https://textilelearner.net/imperfection-index-of-yarns-reasons-for-increasing/.

[24] (2023). How the yarn RKM valaue relates to yarn strength?. https://www.answers.com/Q/How_the_yarn_RKM_valaue_relates_to_yarn_strength.

[25] (2023). RKM: an important parameter of yarn quality. https://www.thefreelibrary.com/RKM%3A+An+important+parameter+of+yarn+quality.-a0301841433.

[26] (2023). Yarn strength and elongation | tensile testing equipment. https://www.testresources.net/applications/test-types/tensile-test/yarn-strength-and-elongation-tensile-testing-equipment/.

[27] Chowdhury M.F., Islam M.N. (2022 Aug 1). Qualitative and statistical analysis of cotton-flax blend yarn. Heliyon.

[28] Pan N., Hua T., Qiu Y. (2001 Nov). Relationship between fiber and yarn strength. Textil. Res. J..

[29] Hassen M.B., Abualsauod E., Halimi M.T., Othman A. (2020 Sep 1). Quality yarn index using AHP and Fuzzy method. Industria Textila.

[30] Viswanathan G., Munshi V.G., Ukidve A.V., Chandran K. (1989 Nov). A critical evaluation of the relationship between fiber quality parameters and hairiness of cotton yarns. Textil. Res. J..

[31] (2023). What IS a box and whisker plot?. https://asq.org/quality-resources/box-whisker-plot#:%7E:text=A%20box%20and%20whisker%20plot%20is%20defined%20as%20a%20graphical,displayed%20in%20the%20same%20graph.

[32] Potter K., Hagen H., Kerren A., Dannenmann P. (2006 Jan).

[33] Marmolejo-Ramos F., Tian T.S. (2010). The shifting boxplot. A boxplot based on essential summary statistics around the mean. Int. J. Psychol. Res..

[34] Grömping U. (2015 Mar). Variable importance in regression models. Wiley interdisciplinary reviews: Comput. Stat..

[35] Nathans L.L., Oswald F.L., Nimon K. (2012 Apr). Interpreting multiple linear regression: a guidebook of variable importance. Practical Assess. Res. Eval..

[36] Ullah A.A., Foisal A.B. (2019 Jun). A review on sustainable textile products from jute and cotton blends. SEU J. Sci. Eng..

[37] Elahi S., Hosen M.D., Islam M., Hasan Z., Helal M.M., Al M.S., Rakin S. (2019). Analysis of physical & chemical properties of cotton-jute blended denim after a sustainable (industrial stone enzyme) wash. Journal of Textile Science and Fashion Technology.

[38] Ullah A.A., Foisal A., Nahar N. (2016). Study on the characteristics of jute-cotton blended fabrics. SEU Journal of Science and Engineering.

